# Supplementary material for: A Global Screen for Assembly State Changes of the Mitotic Proteome by SEC-SWATH-MS
Source: Cell Syst. 2020 Feb 26;10(2):133–155.e6. doi: 10.1016/j.cels.2020.01.001 (PMC7042714; doi:10.1016/j.cels.2020.01.001)

Q9BUT9 | MCRI2_HUMAN | MCRIP2 C16orf14 FAM195A
Monomer MW [kDa]: 17.828 Monomer expected elution fraction: 55

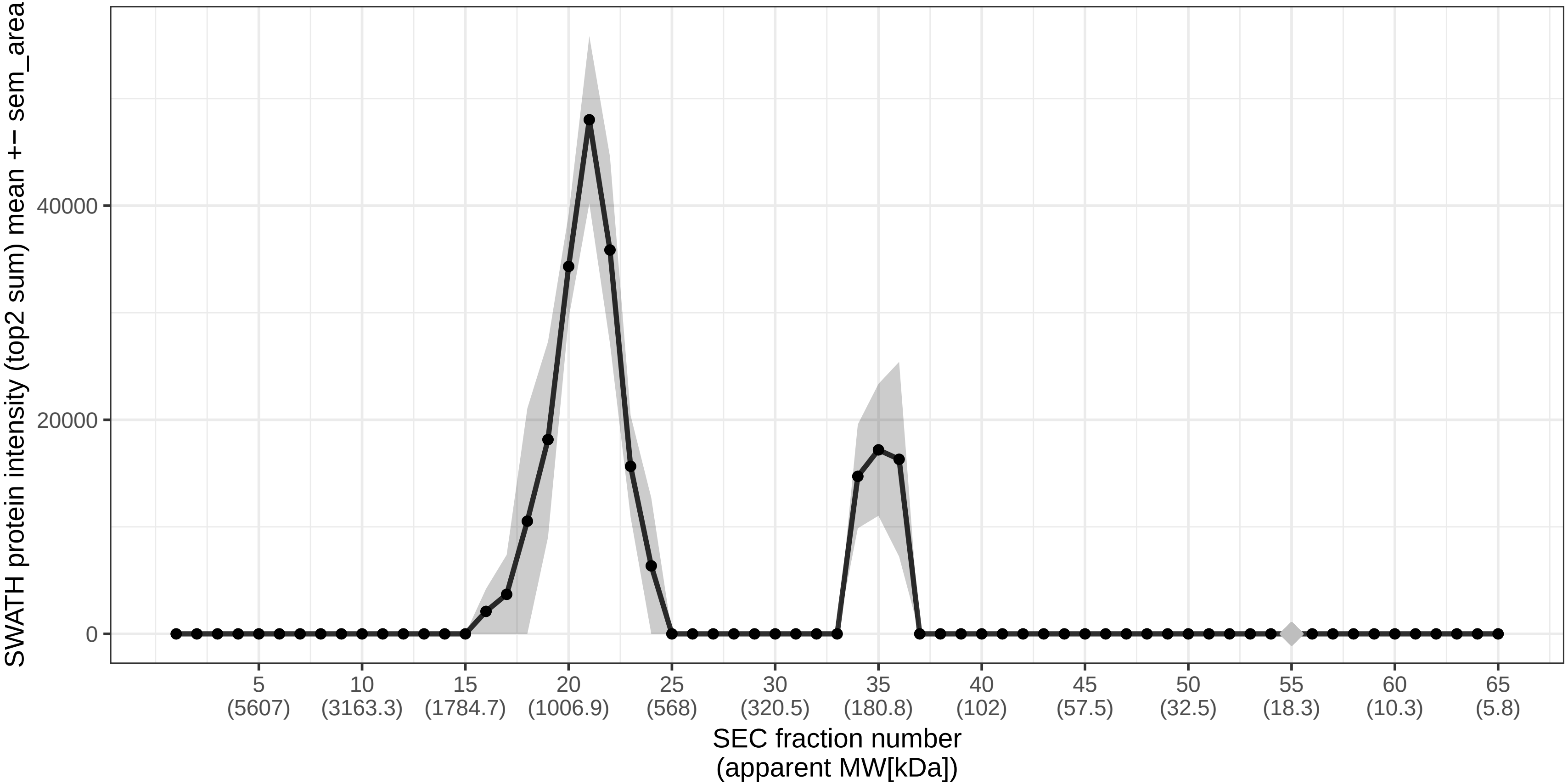

Supplement: Data S1. SEC-SWATH-MS Protein Chromatograms, Related to Figure 1 [file mmc6.zip › SECchrom_Q9BUT9_MCRI2_HUMAN_MCRIP2_C16orf14_FAM195A.pdf]
